# Quack Doctors and Cheap Medical Colleges

**Published:** 1872-01

**Authors:** W. C. Coleman


### QUACK DOCTORS AND CHEAP MEDICAL COLLEGES.

BY W. C. COLEMAN, M. D.

Believing it to be the duty of every true physician, to use his influence against all kinds of quackery and humbuggery, and to use all honorable means in his power for the suppression of the same, I give your readers the following sketch of my experience with a few of the "*Men of Specifics*," who have fallen under my observation.

When returning from the army, in 1865, in the city of Harrisburg, Pa., I met one "Dr. Schultz," who, by some means, learned I had been a medical student before the war.—He appeared extremely anxious to form my acquaintance, and having succeeded, made me the very generous offer of a partnership with him, if I would pay $75, and read with him six months. This offer, made without inquiring how long I had read, or anything about my knowledge of medicine, was overwhelmingly stunning. Yet, I politely informed my liberal friend, that I preferred reading more and attending two or three sessions at some good Medical College, and that I considered his way of affording one a liberal medical education entirely "too cheap to be of any value." Whereupon, he began advocating the great value of the specifics, selected by the "*head men*," and the great simplicity of their practice over all others. I at once agreed to the simplicity of the affair, and left him enjoying its benefits.

A short time after this, while reading medicine at Saltsburg, Pa., a certain "Dr. Simpson" located in the town and put out flaming advertisements, claiming a new and better way of curing disease; and only asked "*an open field and fair play.*" In conversation with him, I learned he had attended "THE PHILADELPHIA UNIVERSITY OF MEDICINE AND SURGERY," and that his term had been so brief, that he could not inform me how many professors there were connected with the College; had forgotten all their names except one, and for some unknown reason was unable to find his tickets. He returned to the same College the next winter, graduated, and early in the Spring re-opened his office and resumed practice. He at once began borrowing money, "put on much style," and, ere long received a *night call* to some unknown section, from which he has not yet returned. He not only left himself, but left the money, rent, board, washing, and other bills, unpaid.

When in Philadelphia, attending lectures, I was twice permitted to be present at the surgical clinic of the "Modern University," where I saw poultices, ointments, and liniments used profusely, to the exclusion of the knife. I also heard one lecture on practice, but the learned professor did everything either to encourage or prevent metastasis, and I failed to see his good points. While in New York, attending College, one Nathaniel Clark of N. H., came in, when the session was about one-fourth advanced, and took out *four tickets.* He and I roomed together, and he generally attended all the lectures, but utterly failed to comprehend any.—When I accused him of a want of application, he confessed—although he had a certificate for one year's reading from his preceptor—that he had only read three months; that he had spent his life as a farmer, a huckster, a painter, a pedler, and a tooth extractor; and that as he had not even a good English education, and was 36 years old, it was simply impossible for him to understand the lectures. The longer he attended the more he became mixed, and several weeks before the session closed, he left in disgust. The next I heard of N. Clark was at Callowhill Medical College, Phil'a., the following winter, and he informed me at the time of writing that he would soon graduate, (and did), after which he would return to some of the N. E. States and practice.

Such are the men turned out from the Philadelphia Eclectic Colleges, and if your readers will bear with me, I will give a little of the practical qualifications of one educated in the West. A few months ago a Dr. Evans located in our town. He claimed to have enjoyed a lucrative practice in Cincinnati, and only left it on account of his wife's health. After being here for some time, he obtained some practice and claimed some success. Things moved slowly and apparently smoothly, until a few weeks ago, when the "eminent Cincinnati Dr." was called to attend an obstetrical case. He responded promptly to the call and soon took his seat at the bedside, and for a time he reported favorable progress. But lo, and behold! what was his consternation when he discovered that "the bladder had *bursted clean through the vaginal wall* and presented the fundus in the vagina." After making great efforts to adjust the displaced organ, he informed the ladies present of the condition of affairs; that he had twice returned the treacherous bladder to its proper place, but it would not remain, and he must now draw off its contents, and *then* he thought he could "put it back." He accordingly armed himself with the largest sized catheter, and, after several unsuccessful attempts to introduce the formidable instrument, claimed that it was impossible for one physician to manage such a case, and rushed off to procure help, in a short time returning with a physician who was anxious to see the case of "*inversion of the bladder;*" but he soon discovered he had seen such cases before, and, although the long catheter was just at hand, he found the index finger was the proper instrument required, and the bladder that had been so troublesome, suddenly disappeared, and the water was not obliged to pass by the circuitous route of the urethra.—The labor soon terminated favorably under the care of the physician who had been called.

Such is the history, in brief, of a few of the learned and profound medical gentlemen, graduates of the cheap, quack schools (?) of Philadelphia. Let the people learn what sort of "Doctors" they are "graduating" every day.

---

FEMALE DRESS.—Female costume is, perhaps, the most expensive result of the fall. No sooner had Eve bitten the apple than she discovered she wanted a dress; and that want has been increasing in intensity and comprehensiveness among her daughters ever since that unfortunate hour.

